# Case Report: A novel *LHFPL3::NTRK2* fusion in dysembryoplastic neuroepithelial tumor

**DOI:** 10.3389/fonc.2022.1064817

**Published:** 2022-12-01

**Authors:** Yanming Chen, Qing Zhu, Ye Wang, Xiaoxiao Dai, Ping Chen, Ailin Chen, Sujuan Zhou, Chungang Dai, Shengbin Zhao, Sheng Xiao, Qing Lan

**Affiliations:** ^1^ Department of Neurosurgery, The Second Affiliated Hospital of Soochow University, Suzhou, China; ^2^ Heath Management Center, The Second Affiliated Hospital of Soochow University, Suzhou, China; ^3^ Department of Pathology, The Second Affiliated Hospital of Soochow University, Suzhou, China; ^4^ Molecular Genetics Laboratory, Suzhou Sano Precision Medicine Ltd., Suzhou, China; ^5^ Pathology and Pathophysiology, Soochow University Medical College, Suzhou, China; ^6^ Department of Pathology, Brigham and Women’s Hospital, Boston, BS, United States

**Keywords:** dysembryoplastic neuroepithelial tumors, NTRK2, LHFPL3, next-generation sequencing, IDH1

## Abstract

Neurotrophic tyrosine receptor kinase (*NTRK*) rearrangements are oncogenic drivers of various types of adult and pediatric tumors, including gliomas. However, *NTRK* rearrangements are extremely rare in glioneuronal tumors. Here, we report a novel *NTRK2* rearrangement in a 24-year-old female with dysembryoplastic neuroepithelial tumor (DNT), a circumscribed WHO grade I benign tumor associated with epilepsy. By utilizing targeted RNA next-generation sequencing (NGS), fluorescence *in situ* hybridization (FISH), reverse transcriptase PCR (RT-PCR), and Sanger sequencing, we verified an in-frame fusion between *NTRK2* and the lipoma *HMGIC* fusion partner-like 3 (*LHFPL3*). This oncogenic gene rearrangement involves 5’ *LHFPL3* and 3’ *NTRK2*, retaining the entire tyrosine kinase domain of *NTRK2* genes. Moreover, the targeted DNA NGS analysis revealed an *IDH1* (p.R132H) mutation, a surprising finding in this type of tumor. The pathogenic mechanism of the *LHFPL3::NTRK2* in this case likely involves aberrant dimerization and constitutive activation of RTK signaling pathways.

## Introduction

The neurotrophic tyrosine receptor kinase (*NTRK*) family includes *NTRK1, NTRK2*, and *NTRK3* which encode neurotrophin receptors TrkA, TrkB, and TrkC, respectively. Fusions involving the *NTRK* family are one of the most common mechanisms of oncogenic Trk activation (1) and occur sporadically in low-grade gliomas and glioblastoma (2). A variety of N-terminal gene fusion partners are described (3), which replace the ligand-binding site of the Trk, resulting in ligand-independent dimerization and phosphorylation of the Trk (4).

We describe here a novel *NTRK2::LHFPL3* fusion in a Dysembryoplastic neuroepithelial tumor (DNT), a low-grade glioneuronal tumor. While the signaling pathways of TrkB are well-studied, not much is known about the *LHFPL3.* Lipoma *HMGIC* fusion partner-like 3 (*LHFPL3*) is a member of the superfamily of tetra-span transmembrane proteins. RNA-Seq expression data from GTEx showed a dominant *LHFPL3* expression in the brain, including amygdala, anterior cingulate cortex, caudate, front cortex, hippocampus, hypothalamus, nucleus accumbens, putamen, and substantia nigra, while its expression is minimal in other non-brain tissues. Two human diseases have been linked to this group of genes. One *LHFP*-like gene is fused to the high mobility group AT-Hook 2 (*HMGA2*) in lipomas (5). Mutations in an *LHFP*-like gene result in deafness (6).

DNTs represent a type of glioneuronal and neuronal tumor (WHO, grade I) in the central nervous system (CNS) (7) and is the second most prevalent CNS neoplasm associated with epilepsy onset (8). DNT was first described by Daumas-Duport in 1988 (9), characterized by a multinodular growth of specific glioneuronal elements, with the columnar architecture of oligodendrocyte-like cells oriented perpendicular to the cortical surface and floating neurons in an abundant mucinous matrix (10, 11). DNT has shown genetic diversity and lacks molecular characteristics (12). Although studies have indicated *FGFR1* mutations are more common in DNTs (10), other genetic alterations were also reported (13). While certain pediatric gliomas have been shown to harbor *NTRK* fusions with frequencies of 5–25% (14), only one case of mixed neuronal-glial tumors (MNGT) with *SPECC1L::NTRK2* fusion was reported (12). Due to the exceeding rare *NTRK* rearrangement in this group of glioneuronal and neuronal tumors, we conducted a comprehensive integrating analysis of imaging, conventional histopathology, and molecular profiling.

## Materials and methods

### Immunohistochemistry

The tumor tissue was fixed by formalin and embedded in paraffin. The embedded tumor tissue was cut into 5μm thick slices. For immunohistochemistry, the primary antibodies were utilized according to the manufacturer’s protocol. The immunohistochemical staining of pan-TRK (ab181560, Abcam, UK), Synaptophysin (36406, CST, USA), CD34 (Kit-0004, MXB, China), NSE (MAB-0791, MXB, China), Olig2 (ab109186, Abcam, UK), ATRX (sc-55584, Santa Cruz, USA), P53 (2527, CST, USA), IDH1 (R132H) (H09, Dianova, Germany), and Ki67 (9449, CST, USA) followed the protocol as the previous report (15).

### Targeted RNA next-generation sequencing

Total RNA from fresh tumor tissue was extracted with TRIzol™ LS Reagent following the manufacturer’s instructions (10296010, ThermoFisher, Invitrogen, USA). 100ng total RNA was utilized for reverse transcription. End repairing and adaptor ligation were performed according to standard NGS protocols (E7771 and E6111, NEB, USA). PCR enrichment was performed using 390 gene-specific primers specific to a group of 63 genes commonly involved in solid tumors, and the enriched PCR products were sequenced in an Illumina NovaSeq 6000 platform (San Diego, USA). Sequencing results were analyzed with SeqNext software (JSI, Germany).

### Fluorescence *in situ* hybridization

FISH was performed on 5µm paraffin slides of tumor tissue with dual-color break-apart probes for *NTRK2* (Betrue, China). A total of 50 interphases were studied. FISH was applied as previously described (16).

### Reverse transcriptase PCR and Sanger sequencing

Total RNA was extracted with TRIzol™ LS Reagent according to the manufacturer’s instructions (10296010, ThermoFisher, Invitrogen, USA). The RNA integrity was evaluated in agarose gel electrophoresis. cDNA was synthesized with random priming and SuperScript™ IV reverse transcriptase (18090050, ThermoFisher, USA). The first PCR was performed as the previous report (17). The primers were specific for *LHFPL3* and *NTRK2* (*LHFPL3*-F: 5’-CTTCAAAGCCGCCTCCTTCTT, *NTRK2*-R: 5’-TCCTGCTCAGGACAGAGGTTA). The first PCR condition was 95°C 3 min for 1 cycle followed by 35 cycles of 95°C 30s, 58°C 60 sec, and 72°C 60 sec, extend 72°C 5 min. 1μL of the first PCR product was utilized for nested PCR with the nested primers (*LHFPL3*-F: 5’-TCCGCTGCCTGCCTTGTGCTT, *NTRK2*-R: 5’-GCTGAACAAATGTGTCTGGC). Nested PCR condition was 95°C 3 min for 1 cycle followed by 40 cycles of 95°C 30s, 58°C 60 sec, and 72°C 60 sec, extend 72°C 5 min. Eventually, Sanger sequencing was performed to confirm the breakpoint on both *LHFPL3* exon2 and *NTRK2* exon16.

## Case presentation

**Figure 1:**
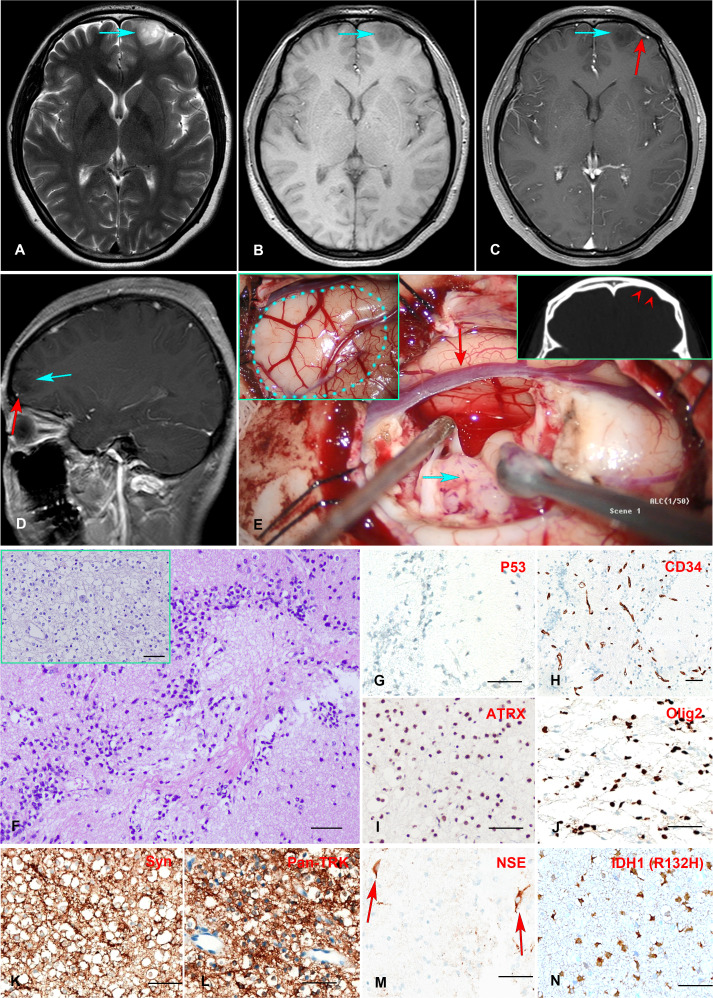
**(A–E)** Preoperative MR imaging and intraoperative stills. T2-weighted MRI revealed an inhomogeneous hyperintense lesion in the left frontal lobe **(A)**. T1-weighted MRI showed a hypointensity of the lesion **(B)**. Enhanced T1-weighted MR images indicated no enhancement of the mass, with a vessel (red arrow) underneath the tumor **(C, D)**. The intraoperative stills revealed the tumor was regionally confined in a lobe. The dotted blue line in the panel delineated the region of the tumor. The red arrowheads indicate left frontal bone deformation and thinning caused by long-term tumor compression**(E)**. **(F–N)** Histopathology and immunohistochemistry staining of the Formalin-fixed paraffin-embedded (FFPE) tissue tumor tissue sections. H&E staining revealed numerous round monotonous oligodendrocyte-like cells, with neurons floating in the matrix. The multinodular glial architecture was also observed (inserted) **(F)**. Immunohistochemical staining indicates negative P53-negative expression **(G)**, scattered positive CD34 expression in tumor cells in addition to vascular endothelial cells **(H)**, positive ATRX expression **(I)**, positive Olig2 expression **(J)**, positive Syn and Pan-TRK expression **(K, L)**, positive NSE expression in neurons **(M)**, and positive mutant IDH1 (R132H) expression **(N)**. Scale bar = 50μm.

A 24-year-old female presented with a history of more than a decade of recurrent and unprovoked seizures. T2 weighted magnetic resonance imaging (MRI) of the brain showed an inhomogeneous hyperintense lesion located in the left frontal lobe (**Figure 1A**), measuring 2.0 × 1.5 × 1.7 cm in dimension, while T1 weighted MR image revealed hypo-intensity without peri-tumoral edema (**Figure 1B**). Contrast-enhanced MRI exhibited no significant enhancement (**Figures 1C, D**).

The patient received extensive tumor resection after careful preoperative planning. The tumor was soft and confined in the lobe (**Figure 1E**). A postoperative MRI confirmed complete resection of the tumor. The fresh tumor tissues were sent for histology and molecular profiling analysis. H&E staining revealed a multinodular glial element, and a honeycomb appearance of oligodendrocyte-like cells was observed, with neurons floating in the matrix (**Figure 1F**). Immunohistochemistry analysis showed positive ATRX nuclear stain and negative P53 expression (**Figures 1G, I**), consistent with the genomic analysis showed wild-type *ATRX* and *TP53*. Scattered CD34 expression was seen in tumor cells in addition to vascular endothelial cells (**Figure 1H**), which was consistent with a previous report showing frequent CD34 expression in mixed or diffuse DNT (18). Tumor cells also expressed synaptophysin (Syn) (**Figure 1K**), oligodendrocyte transcription factor 2 (Olig2) (**Figure 1J**), and Ki-67 positivity less than 5%. Neuron-specific enolase (NSE) expression was observed in the neurons as expected (**Figure 1M**). Taken together, the histopathological and molecular pathological profiles were consistent with a complex subtype DNT. Moreover, Anti-Pan TRK antibody staining revealed a strong signal in tumor cells, consistent with the TrkB rearrangement (**Figure 1L**).

**Figure 2:**
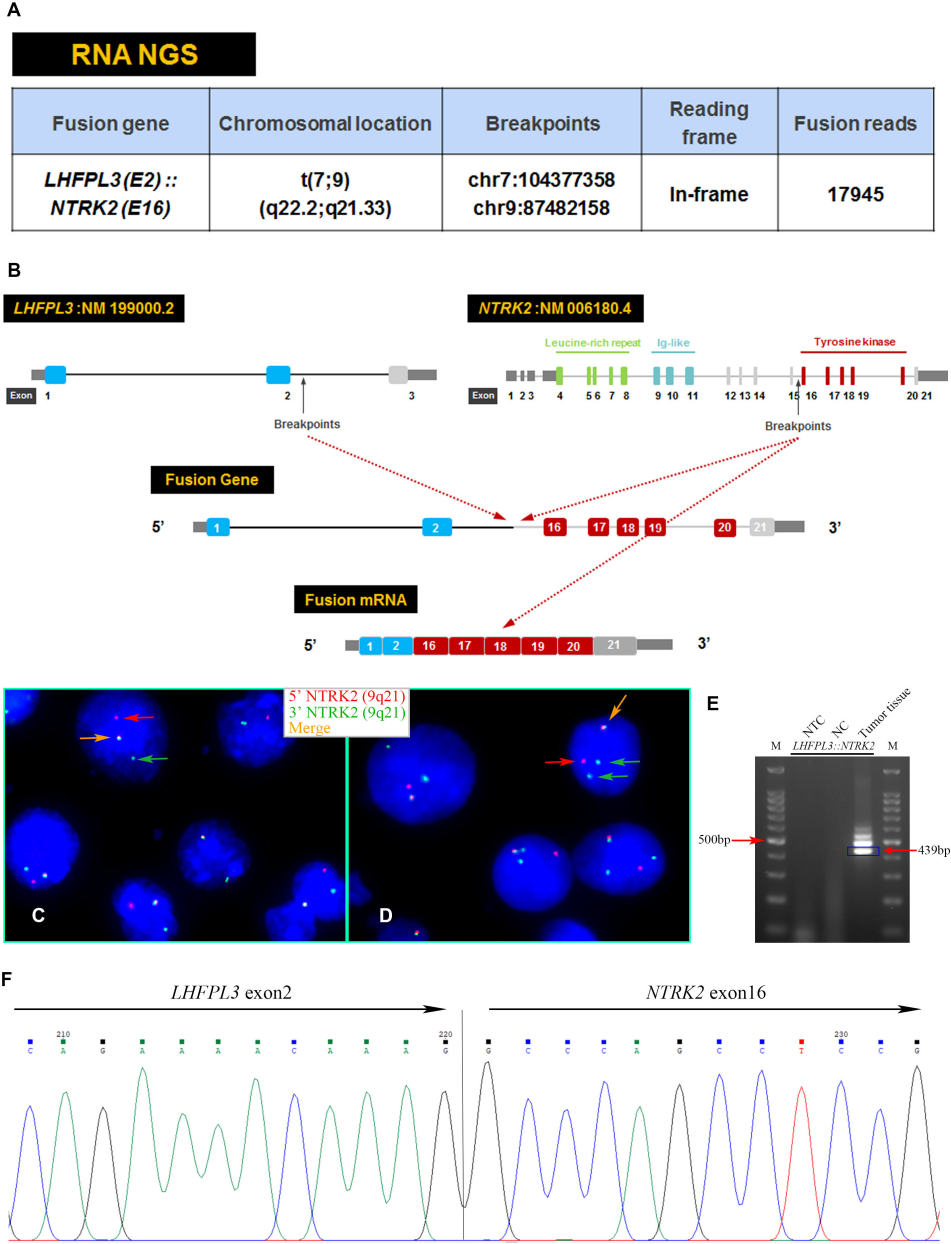
Characterization of the *LHFPL3::NTRK2* fusion. **(A)** Targeted RNA NGS revealed an *LHFPL3::NTRK2* fusion transcript with an intact reading frame. **(B)** A schematic diagram showed the breakpoints of *LHFPL3* and *NTRK2*, with the fused gene retaining the tyrosine kinase domain of the *NTRK2*. **(C, D)** FISH with an *NTRK2* split-apart probe showed the separation of the 5’ *NTRK2* (Red) from the 3’ *NTRK2* (Green), consistent with the *NTRK2* gene rearrangement. **(E)** RT-PCR confirmed the expression of *LHFPL3::NTRK2* fusion transcript. **(F)** Sanger sequencing of the PCR product confirmed the breakpoints of *LHFPL3* and *NTRK2* genes. NTC, No Template Control; NC, Negative Control.

An RNA NGS assay with 390 ‘bait’ probes targeting 63 genes commonly rearranged in solid tumors revealed an *LHFPL3::NTRK2* transcript (**Figure 2A**). The *LHFPL3::NTRK2* fusion gene contained the first two exons of *LHFPL3* and the last 6 exons of *NTRK2*, starting at exon 16 (**Figure 2B**). FISH analysis with a break-apart *NTRK2* probe confirmed the *NTRK2* rearrangement in tumor cell touch preparation (**Figures 2C, D**).

**Figure 3:**
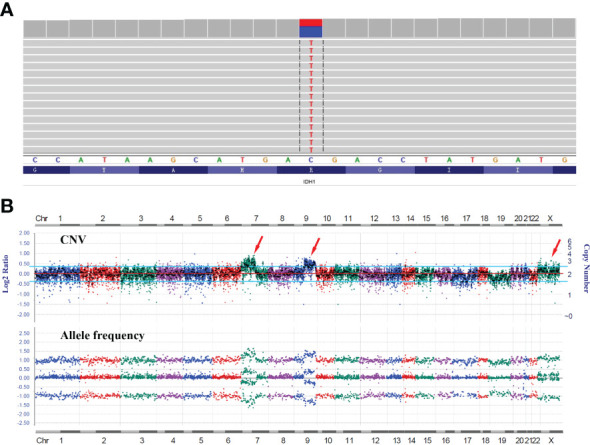
DNA NGS results of the tumor. **(A)** IGV image of the *IDH1* mutation (p.R132H). **(B)** Copy number variation (CNV) analysis showed gains of the chromosomes 7p, 9q, and X with no evidence of 1p/19q co-deletion.

RT-PCR analysis of tumor RNA with primers specific for *LHFPL3* and *NTRK2* showed 3 bands. The dominant band (**Figure 2E**) was Sanger sequenced and was identical to the transcript obtained from the RNA NGS. The other 2 minor bands were not sequenced, likely from differential splicing. A targeted DNA NGS (**Figure 2F**) analysis revealed an isocitrate dehydrogenase 1 (*IDH1*) mutation (p.R132H) (**Figure 3A**) and copy number gains of the chromosomes 7p, 9q, and X (**Figure 3B**). Immunohistochemistry analysis also confirmed the expression of mutant *IDH1* (R132H) (**Figure 1N**). *IDH1* mutations are often seen in diffuse gliomas including astrocytoma or oligodendroglioma but are rarely reported in DNTs (19). Other mutations, including *ATRX* and *TP53* mutations in astrocytoma and chromosome 1p/19q co-deletion in oligodendroglioma, were not observed in this tumor.

## Discussion

A variety of genomic alterations have been reported in DNTs, although diagnostically specific changes are not established. Nevertheless, fibroblast growth factor 1 receptor (*FGFR1*) has been frequently activated in DNTs (10, 20). Among *FGFR1* alterations, internal tandem duplication (ITD) of the tyrosine kinase domain (TKD) is the most common mutation, which was reported in 40~60% of DNTs. In addition, hotspot missense mutations of *FGFR1* were also reported. Other genomic alterations include *BRAF* p.V600E and copy number chromosome gains (21). The frequency of *BRAF* alterations in DNTs remains inconclusive among different studies (22, 23), with several studies failing to identify *BRAF* alterations in their DNTs cohorts (20, 24).

*NTRK* rearrangements in glioneuronal tumors were extremely rare. *STRN1::NTRK2* and *ARHGEF2::NTRK1* were reported in a single case of malignant glioneuronal tumor, respectively (25, 26). Alvarez-Breckenridge (27) described a case of low-grade glioneuronal tumor with *BCAN::NTRK1* fusion. Surrey (12) reported a case of mixed neuronal-glial tumors (MNGT) with *SPECC1L::NTRK2* fusion. Torre et al. reported a cohort of gliomas harboring *NTRK* fusions, most infantile or adult cases were histologically high-grade (89.7%, 26/29), while pediatric cases demonstrated high-grade histology (15.4%, 2/13) were rare. The median follow-up period after diagnosis was 23 months. During the follow-up period, 57.0% of the cases suffered tumor recurrence or progression (28).

*IDH1/2* mutations are rare in DNTs, different from low-grade diffuse gliomas. In a cohort of 100 DNTs, 3 *IDH1* mutations were observed (19). Jayapalan (29) reported a rosette-forming glioneuronal tumor with *IDH1* mutation, which recurred *in situ* and progressed to glioblastoma 6 years after partial resection. Authors speculated that *IDH1* mutation may lead to the malignant transformation of this type of benign glioneuronal tumor. Nevertheless, the biological and prognostic implications of *IDH1/2* mutations in DNTs remain to be clarified.

In conclusion, we report a rare DNT with a novel *LHFPL3::NTRK2* fusion and an *IDH1* mutation. These findings provide additional evidence for a heterogeneous genomic profile of DNTs. Further functional evaluation of the *LHFPL3::NTRK2* fusion oncoprotein will not only detail the oncogenic signaling mechanism but also shed light on the cellular function of *LHFPL3*, a CNS-specifically expressed gene with no studies.

## Data availability statement

The original contributions presented in the study are included in the article/Supplementary Material. Further inquiries can be directed to the corresponding author.

## Ethics statement

This study was reviewed and approved by the Ethics Committee of the Second Affiliated Hospital of Soochow University. Written informed consent to participate in this study was provided by the participant herself. 

## Author contributions

YC designed this study. XD, PC, SuZ, and ShZ performed histopathological and molecular pathological examinations. XD and SX were responsible for the pathological diagnosis. QZ, YW, AC, and CD supported clinical data. QL and SX guided this work. All authors contributed to the article and approved the submitted version.

## Funding

This study was funded by the National Natural Science Foundation of China (81602183).

## Conflict of interest

Authors PC, SuZ, and ShZ were employed by Suzhou Sano Precision Medicine Ltd.

The remaining authors declare that the research was conducted in the absence of any commercial or financial relationships that could be construed as a potential conflict of interest.

## Publisher’s note

All claims expressed in this article are solely those of the authors and do not necessarily represent those of their affiliated organizations, or those of the publisher, the editors and the reviewers. Any product that may be evaluated in this article, or claim that may be made by its manufacturer, is not guaranteed or endorsed by the publisher.
